# Antidiabetic and antihyperlipidemic activities of *Phyllanthus emblica L*. extract *in vitro* and the regulation of Akt phosphorylation, gluconeogenesis, and peroxisome proliferator-activated receptor α in streptozotocin-induced diabetic mice

**DOI:** 10.29219/fnr.v67.9854

**Published:** 2023-10-13

**Authors:** Shin-Ming Huang, Cheng-Hsiu Lin, Wen-Fang Chang, Chun-Ching Shih

**Affiliations:** 1 Department of Gastroenterology, Jen-Ai Hospital, Dali Branch, Taichung City, Taiwan; 2 Department of Internal Medicine, Fengyuan Hospital, Ministry of Health and Welfare, Taichung City, Taiwan; 3 Department of Cardiology, Jen-Ai Hospital, Taichung City, Taiwan; 4 Department of Nursing, College of Nursing, Central Taiwan University of Science and Technology, Taichung City, Taiwan

**Keywords:** diabetes, streptozotocin, Phyllanthus emblica L, antihyperlipidemic, gluconeogenesis, insulin-expressing β cells

## Abstract

**Background:**

The fruits of *Phyllanthus emblica* L. are high in nutrients and have excellent health care function and developmental value. There are many management strategies available for diabetes and hyperlipidemia. Nevertheless, there is a lack of an effective and nontoxic drug.

**Objective:**

The present study was designed to first screen four extracts of *P. emblica* L. on insulin signaling target gene expression levels, including glucose transporter 4 (GLUT4) and p-Akt/t-Akt. The ethyl acetate extract of *P. emblica* L. (EPE) exhibited the most efficient activity among the four extracts and was thus chosen to explore the antidiabetic and antihyperlipidemic activities in streptozotocin (STZ)-induced type 1 diabetic mice.

**Design:**

All mice (in addition to one control (CON) group) were administered STZ injections (intraperitoneal) for 5 consecutive days, and then STZ-induced mice were administered EPE (at 100, 200, or 400 mg/kg body weight), fenofibrate (Feno) (at 250 mg/kg body weight), glibenclamide (Glib) (at 10 mg/kg body weight), or vehicle by oral gavage once daily for 4 weeks. Finally, histological examination, blood biochemical parameters, and target gene mRNA expression levels were measured, and liver tissue was analyzed for the levels of malondialdehyde (MDA), a maker of lipid peroxidation.

**Results:**

EPE treatment resulted in decreased levels of blood glucose, HbA1C, triglycerides (TGs), and total cholesterol and increased levels of insulin compared with the vehicle-treated STZ group. EPE treatment decreased blood levels of HbA1C and MDA but increased glutathione levels in liver tissue, implying that EPE exerts antioxidant activity and could prevent oxidative stress and diabetes. The EPE-treated STZ mice displayed an improvement in the sizes and numbers of insulin-expressing β cells. EPE treatment increased the membrane expression levels of skeletal muscular GLUT4, and also reduced hepatic mRNA levels of glucose-6-phosphatase (G6Pase) and phosphoenolpyruvate carboxykinase thereby inhibiting hepatic gluconeogenesis. This resulted in a net glucose lowering effect in EPE-treated STZ mice. Furthermore, EPE increased the expression levels of p-AMPK/t-AMPK in both the skeletal muscle and liver tissue compared with vehicle-treated STZ mice. EPE-treated STZ mice showed enhanced expression levels of fatty acid oxidation enzymes, including peroxisome proliferator-activated receptor α (PPARα), but reduced expression levels of lipogenic genes including fatty acid synthase, as well as decreased mRNA levels of sterol regulatory element binding protein 1c (SREBP1c), apolipoprotein-CIII (apo-CIII), and diacylglycerol acyltransferase-2 (DGAT2). This resulted in a reduction in plasma TG levels. EPE-treated STZ mice also showed reduced expression levels of PPAR γ. This resulted in decreased adipogenesis, fatty acid synthesis, and lipid accumulation within liver tissue, and consequently, lower TG levels in liver tissue and blood. Furthermore, EPE treatment not only displayed an increase in the Akt activation in liver tissue, but also in C2C12 myotube in the absence of insulin. These results implied that EPE acts as an activator of AMPK and /or as a regulator of the insulin (Akt) pathway.

**Conclusions:**

Taken together, EPE treatment exhibited amelioration of the diabetic and hyperlipidemic state in STZ-induced diabetic mice.

## Popular scientific summary

- Treatment with ethyl acetate extract of *P. emblica* L. (EPE) significantly increased blood insulin levels and lowered blood glucose, triglyceride, total cholesterol, and HbA1c levels in streptozotocin (STZ)-induced diabetic mice.
- Treatment with EPE-regulated critical pathways associated with promoting glucose transport in skeletal muscles, suppressing hepatic glucose production, promoting lipid metabolism, including AMP-activated protein kinase (AMPK) activation and lipolysis, and inhibiting fatty acid synthesis in liver tissues.

Diabetes mellitus is an important health issue. Epidemiological studies have indicated that the prevalence of type 1 diabetes mellitus (T1DM) patients is approximately 5–10% of all diabetic cases (1). T1DM is characterized by persistent hyperglycemia, and histological examination has shown that there are numerous destructive beta (β) cells within the pancreas (2), through pathophysiology that involves destruction of pancreatic β-cells. Individuals with T1DM have very little insulin produced by the islets of Langerhans (including β cells). T1DM often occurs in young individuals and is an autoimmune disease that arises from the selective and progressive loss of insulin-producing β cells (2, 3). Insulin is stored in the granules of the β cell. Insulin is secreted into the blood to maintain normal blood glucose levels. Diabetes mellitus is diagnosed by circulating levels of glucose or HbA1C (4). Therapeutic regimens for T1DM involve insulin treatment; nevertheless, insulin needs to be injected.

**Fig. 1:**
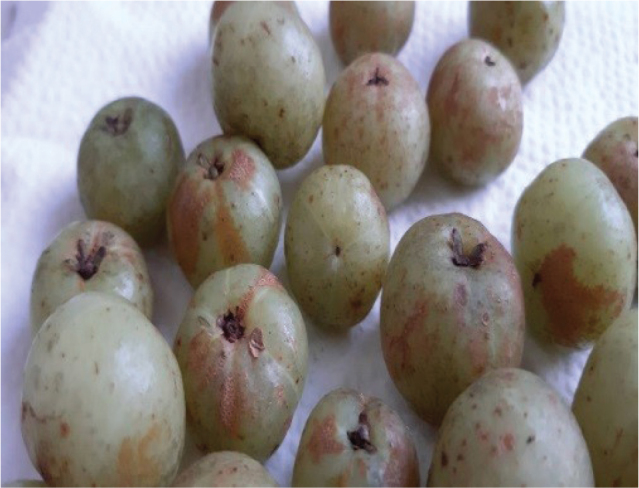
The fruits of *Phyllanthus emblica* L.

*Phyllanthus emblica* L. (Fig. 1) is widely distributed in tropical and subtropical regions, including Taiwan. This plant has recently been included among three plants with health benefits that are worthy of promotion and cultivation by the United Nations Organization for Health and Well-being. More recently, the Taiwanese local association (the Agricultural Committee Miaoli District Agricultural Improvement Field in Taiwan) aimed to improve the health-related and industrial applications of this plant as a starting point to initiate its breeding activity. The appearance of *P. emblica* fruit is yellow green, and its taste is slightly bitter. The fruit’s constituents have been shown to have functional activities that promote health. Fruits of *P. emblica* contain ascorbic acid, phenols (consisting of ellagic acid and gallic acid), quercetin, proanthocyanidins, and ellagitannins (5–8). There is evidence that the major functional activity of *P. emblica* is due to its excellent antioxidant activity (5, 7, 9–12). Fruits of *P. emblica* have different pharmacological activities, including gastroprotective (13) and antitussive activities (14).

The polyphenol components of *P. emblica*, including gallic and ellagic acid, have significant antioxidant activity (15), and *P. emblica* extract has been shown to have numerous beneficial effects (16). The leaf of this plant has antioxidant and antifibrotic activity within the lung (17).

*P. emblica* L. extract has been shown to exhibit blood glucose-lowering properties due to its antioxidant activity in STZ-induced diabetic rats (9, 11). Nevertheless, the molecular mechanism remains to be clarified.

There are two principal mechanisms involved in promoting translocation of glucose transporter 4 (GLUT4) to the plasma membrane including insulin signaling through the phosphatidylinositol 3’ kinase (PI3-kinase)/Akt pathway and the AMP-activated protein kinase (AMPK) pathway (18). The two major pathways that converge to stimulate glucose uptake are insulin signaling via the Akt and AMPK pathways. Insulin stimulates glucose uptake *via* promotion of the net translocation of GLUT4 to skeletal muscle membranes and adipose cells (19, 20). AMPK is a phylogenetically conserved intracellular fuel sensor that regulates fatty acid oxidation and lipid synthesis in most cells (21). It is well-known that there are crucial rate-limiting gluconeogenic enzymes in liver tissues: the phosphoenolpyruvate carboxykinase (PEPCK) enzyme and glucose-6-phosphatase (G6Pase) enzyme (22). Diabetic animals exhibit enhanced hepatic G6Pase activity (22).

The present study investigated the functional activities of ethyl acetate extract of *P. emblica* L. (EPE) using a mouse model of STZ-induced diabetes. The chemical STZ is a widely used diabetogenic agent (23–26) and can produce reactive oxygen species (ROS) (27). The dosage of STZ is known to affect the degree of pancreatic β-cell destruction (28, 29), and several STZ injections at low dosages are usually utilized to create a T1DM animal model with a 70% reduction in islets in the pancreas (28, 29). Thus, this model was chosen in this study to examine the preventive activity of EPE in diabetic mice compared with that of other clinical drugs, namely, an oral hypoglycemic sulfonylurea, glibenclamide (Glib) (Glib is not recommended for the treatment of T1DM since it can directly stimulate insulin release from β cells) (30), and a hypotriglyceridemic drug, fenofibrate (Feno), which was the drug chosen for the treatment of hypertriglyceridemia and is a peroxisome proliferator-activated receptor (PPAR) α agonist (31, 32).

Our present study was designed to examine whether EPE could have the potential to protect from T1DM and displays an improvement in pathophysiology of T1DM and the signaling pathway including amelioration of destruction of pancreatic β-cells and oxidant damage, and two signaling pathways, that is, the insulin-Akt pathway and the phosphorylation of AMPK pathway, which contribute to the increase in the levels of membrane GLUT4 protein and promote glucose uptake from the blood to intracellular sites. Thus, we evaluated whether treatment with the ethyl acetate EPE could be effective in lowering glucose concentrations in diabetic mice. Although much has been learned about this metabolic disorder, the precise mechanism of this disease and the pathophysiology of EPE treatment remain unknown. Moreover, the downregulated gene expression of antidiabetic and anti-lipogenic genes was analyzed in the livers and skeletal muscle of EPE-treated diabetic mice.

## Materials and methods

### Fruit materials and preparation of ethyl acetate extract of Phyllanthus emblica L.

Fruits of *P. emblica* L. (Fig. 1) were purchased from Taiwan Miaoli County, Ogan Marketing Corporation. The processing of fruit material and preparation of EPE were conducted as described in our previous study (33). These fruits were identified by China Medical University, Taiwan, and the voucher specimen (CMPF385) was replaced. First, 7.3 kg of *P. emblica L*. fruit (Fig. 1) was dried and pulverized into 1.34 kg of fine powder. The fine powder was extracted with 6.3 L of methanol three times at room temperature and concentrated under vacuum, and then the crude methanolic extract (331.86 g) was obtained. The crude methanolic extract was subjected to three rounds of suspension in H_2_O and partitioning with EtOAc, followed by concentration under reduced pressure, and then the H_2_O fraction (323.71 g) and the EtOAc fraction (47.28 g) were obtained. The EtOAc fraction was employed for the animal study as described in our previous study (33).

### Analysis of Akt phosphorylation in vitro

Cell lines (mouse myoblast; BCRC number 60083) were utilized as described in a previous study (29, 34, 35). Treatments with one of the four extracts or with insulin are depicted in previous studies (29, 34, 35). The four extracts of *P. emblica* were an ethyl acetate extract (EPE), butanol extract (BPE), methanol extract (MPE), and water extract (WPE).

### Animal study

Mice (C57BL/6J) treatments are described in previous studies (29, 34, 35) and were conducted under the guidepost of the school and according to the Animal Ethics Committee (approval no. 109-CTUST-001). Forty-two male mice were obtained from the National Laboratory Animal Breeding Center at 4 weeks of age. Animals were maintained in cages in a room and acclimatized to standard laboratory conditions. After 1 week, one group (*n* = 6) of control (CON) mice (Group I: normal mice) was given identical volumes of vehicle orally, while the others (*n* = 36) were administered STZ injections (intraperitoneal; i.p.) for five consecutive days as described in previous studies (29, 34, 35). Briefly, citrate buffer was first prepared. The STZ preparation method involved dissolving 9.45 g of citric acid in 1 L of ddH_2_O to make the citrate buffer (0.05 mole/L), adjusted to pH = 4.5 (29, 34, 35), which was stored away from light and refrigerated. Then, 66 mg of STZ was dissolved in 120 mL of citrate buffer. STZ mice gradually developed hyperglycemia. Mice were defined as having diabetes if they had blood glucose concentrations above 250 mg/dL after fasting (29, 34, 35). If the blood levels of glucose were not above 250 mg/dL, the mouse was treated with STZ (i.p.) once the next day. After 1 week, successfully STZ-induced diabetic animals (*n* = 36) were randomly subgrouped into the following six groups: Group II – STZ CON group, diabetic mice given the same volume of distilled water orally; Groups III, IV, and V – Diabetic mice orally given EPE at 100, 200, or 400 mg/kg (which are presented as EPE1, EPE2, and EPE3, respectively); and Groups VI and VII – Diabetic mice orally given either glibenclamide or fenofibrate (10 and 250 mg/kg, respectively). The CON/treatment groups showed the following presentations:

Control: Normal;

STZ: Diabetic Control;

STZ+ EPE1: Diabetic +Treatment 1 (low dosage);

STZ+ EPE2: Diabetic +Treatment 2 (middle dosage);

STZ+ EPE3: Diabetic +Treatment 3 (high dosage);

STZ+ Glib: Diabetic + comparative drug 1 (anti-diabetic drug);

STZ+ Feno: Diabetic + comparative drug 2 (anti-hyperlipidemic drug).

Information about sample calculation and determination of concentrations used in treatment groups involves the following steps. To make the solution of high dosage 400 mg/body weight of EPE, for 100 g body weight mice, we used 40 mg of EPE and vehicle (ex: carboxymethylcellulose; CMC) were added to make the total volume of the solution up to 1 mL, that is, 4 g EPE were dissolved in 0.5% CMC to make the total volume of the solution to 100 mL. Whenever treatment with EPE was conducted, we gave EPE to the mice on the basis of the body weight of the mice on that day (if body weight is 30 g, we gave 0.3 mL EPE to the mice).

Calculation and preparation of the solution of high dosage EPE (400 mg/g body weight):

40 mg/100 g body weight –> dissolved in 1 mL solvent, that is,

40: 1 = X mg: 100 mL,

X = 40 × 100 (mg),

X = 4,000/1,000 (g),

X = 4 (g), that is,

4 g EPE–> dissolved in solvent (ex: CMC) to make up the total volume of the solution to 100 mL.

Treatments were given orally once daily in the morning for 4 weeks. After 10 h of fasting, blood was harvested from the retro-orbital sinuses of mice for assessment of glucose concentrations. All treatments (such as the collection of peripheral organs and analysis of biochemical parameters and adipocytokines) were administered as described in previous studies (29, 34, 35).

### Morphological and immunohistochemical analysis

The morphological procedure was conducted as described in previous studies (29, 34, 35). Insulin-expressing β cells were immunohistochemically (IHC) stained brown, and glucagon-expressing α cells were stained green in the pancreatic islets as described in previous studies (29, 34, 35).

### Relative mRNA levels and Western blotting assays

The relative hepatic mRNA levels of G6Pase, PEPCK, apo C-III, sterol regulatory element binding protein 2 (SREBP2), diacylglycerol acyltransferase-2 (DGAT2), SREBP1c, and β-actin were evaluated by the 2700 GeneAmp™ PCR System (Applied Biosystems (ABI), Waltham, MA, USA), and the primers are depicted in Table 1 (29, 34, 35). The livers and skeletal muscles were immediately removed and quickly homogenized with RIPA buffer (Sigma, St. Louis, MO, USA) prior to Western blotting (29, 34, 35). The expression levels of target genes were assessed by Western blotting as demonstrated in previous reports (27, 32, 33) using the following antibodies: anti-phospho-Akt (Ser^473^) (no. 9271), anti-total-Akt (no. 9272), anti-p-AMPK (Thr^172^) (no. 2535), and anti-total-AMPK (no. 5831) from Cell Signaling Technology (Beverly, USA); anti-FoXO1 (forkhead transcription factor forkhead box O1) (no. ab39670), anti-PPARα (no. ab61182), anti-PPARγ (no. ab209350), anti-FAS (fatty acid synthase) (no. ab128856), and anti-β-actin (no. ab8227) from Abcam (Cambridge, CB2 0AX, UK); and goat anti-mouse IgG coupled to HRP secondary antibody (Jackson Lab., Inc., West Grove, USA) (29, 34, 35). Finally, these results were detected using chemiluminescence kits (Amersham Biosciences ECL^TM^, Buckinghamshire, UK).

**Table 1:** Primers used in the present study

Gene	Accession number	Forward primer and reverse primer	PCR product (bp)	Annealing temperature (°C)
Liver				
PEPCK	NM_011044.2	F: CTACAACTTCGGCAAATACC R: TCCAGATACCTGTCGATCTC	330	52
G6Pase	NM_008061.3	F: GAACAACTAAAGCCTCTGAAAC R: TTGCTCGATACATAAAACACTC	350	50
apo-CIII	NM_023114.3	F: CAGTTTTATCCCTAGAAGCA R: TCTCACGACTCAATAGCTG	349	47
SREBP1c	NM_011480	F: GGCTGTTGTCTACCATAAGC R: AGGAAGAAACGTGTCAAGAA	219	50
DGAT2	NM_026384.3	F: AGTGGCAATGCTATCATCATCGT R: AAGGAATAAGTGGGAACCAGATCA	149	50
SREBP2	AF289715.2	F: ATATCATTGAAAAGCGCTAC R: ATTTTCAAGTCCACATCACT	256	47
β-actin	NM_007392	F: TCTCCACCTTCCAGCAGATGT R: GCTCAGTAACAGTCCGCCTAGA	99	55

apo-CIII = apolipoprotein-CIII.

### Chemical and HPLC analysis

The analyses were conducted on a HITACHI high-performance liquid chromatographic (HPLC) L-5000 system equipped with a degasser, pumps, and a photodiode array (PDA) detector linked to a PC computer running the software program HPLC LACHROM.

### Determination of phenolic compounds

The preparation of the EPE procedure was described in a previous report (33). The mobile phase contained acetonitrile (solvent A) and acidified water with trifluoroacetic acid (0.05%, solvent B). Polyphenolic compounds are natural antioxidants that play major functional roles in plants by scavenging free radicals (33). Polyphenolic compounds can be divided into two broad categories: flavonoids and phenolic acids. This study was designed to examine whether polyphenolic compounds (including gallic acid, chebulagic acid, and ellagic acid) were found in the EPE fraction.

### MDA, GSH-Px, SOD, and GSH analyses in liver tissue

The liver tissue was homogenized on ice in 300 μL of malondialdehyde (MDA) lysis buffer (with 3 μL of BHT (100×) and then centrifuged (13,000 × *g*, 10 min) to remove insoluble material. Lipid peroxidation of the plasma was determined with a Lipid Peroxidation (MDA) Colorimetric/Fluorometric Assay Kit (BioVision; CA, U.S.A.). Glutathione peroxidase (GSH-Px) activity in plasma was measured using the Cayman (MI, U.S.) assay kit and superoxide dismutase (SOD) in the liver tissue was determined with a SOD Assay Kit-WST (Dojindo; MD, U.S.A.). GSH of liver tissues were determined with GSSH/GSH Quantification Kit (Dojindo; MD, U.S.A.) according to the manufacturer’s instructions.

### Statistical analysis

These results were calculated using SPSS software employing one-way ANOVA with Dunnett’s multiple range tests. The results are presented as the mean and standard error. A *p* value less than 0.05 was considered significant.

## Results

### Expression of phosphorylated Akt in vitro

**Fig. 2:**
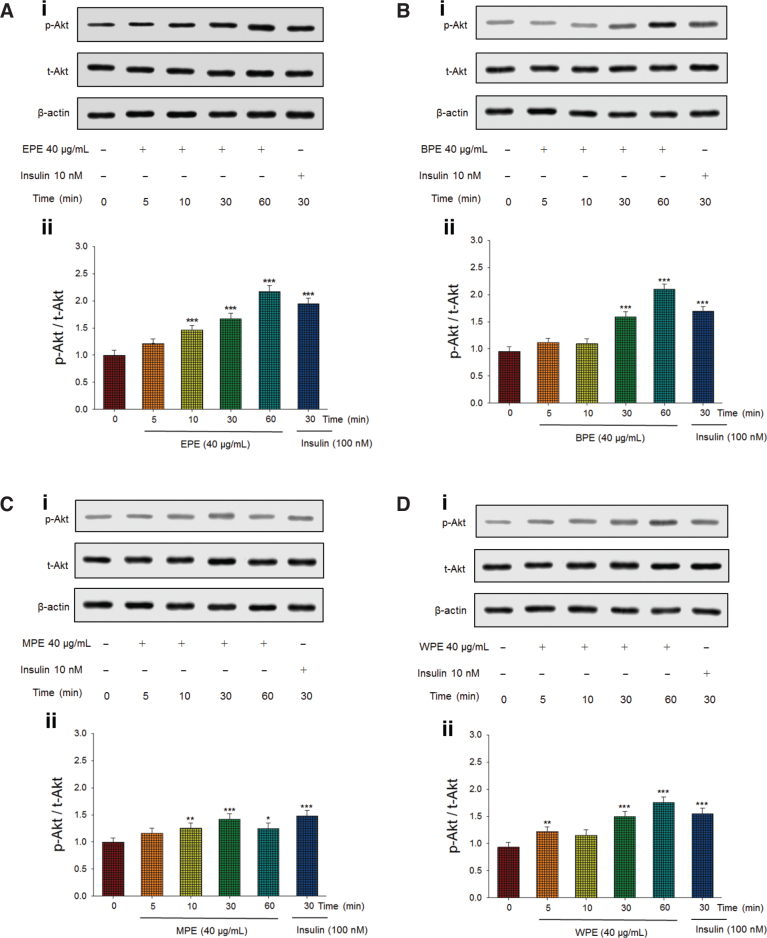
Four extracts of *Phyllanthus emblica* L. activate Akt signaling pathways. Four extracts including (A) ethyl acetate extract of *P. emblica* L. (EPE), (B) butanol extract of *P. emblica* L. (BPE), (C) methanol extract of *P. emblica* L. (MPE), and (D) water extract of *P. emblica* L. (WPE) were analyzed via Western blotting for phospho-Akt (p-Akt) and total-Akt (t-Akt). (1) Representative images; Akt activation is assessed in C2C12 cells, and administered 40 μg/mL EPE for the individual duration (5–60 min); (2) The ratios of p-Akt to t-Akt form were expressed as Akt activation. *P < 0.05, **P < 0.01, or ***P < 0.001 compared to the 0 min group. All values are means ± SE (*n* = 3).

As shown in Fig. 2A, treatment with the EPE significantly increased p-Akt/t-Akt expression, which represents Akt activation, at 10, 30, and 60 min and reached maximum levels at 60 min, which is comparable to insulin action. Figure 2B shows that treatment with the butanol extract of *P. emblica* L. (BPE) significantly enhanced the expression of p-Akt/t-Akt at 30 and 60 min. Figure 2C shows that the methanol extract of *P. emblica* L. (MPE) significantly increased p-Akt/t-Akt expression at 10, 30, and 60 min. Figure 2D shows that the water extract of *P. emblica* L. (WPE) treatment significantly enhanced p-Akt/t-Akt expression at 30 and 60 min.

### Final body weight and relative organ weights

**Fig. 3:**
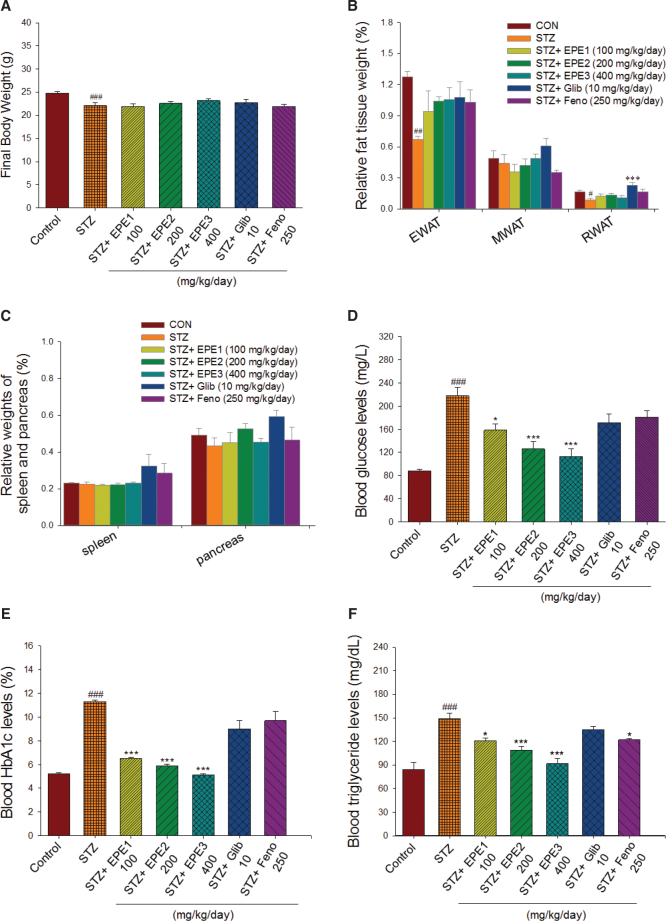

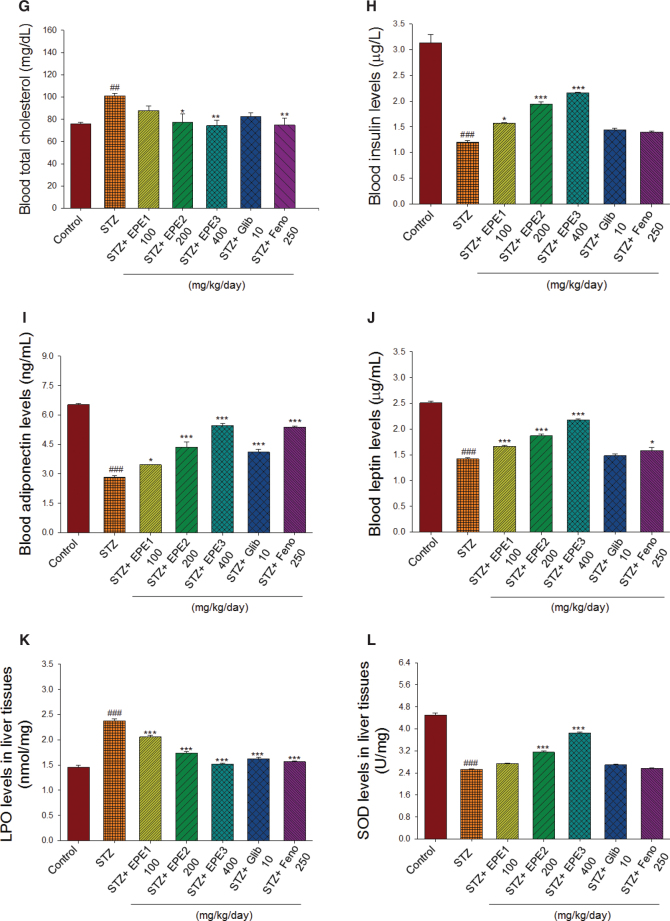

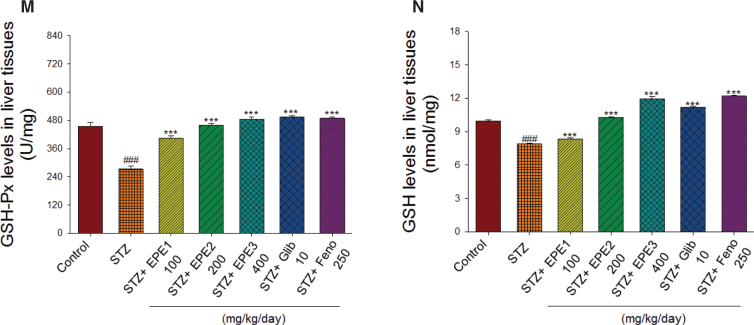
Effects of ethyl acetate extract of *Phyllanthus emblica* L. (EPE) in streptozotocin (STZ)-induced diabetic mice on (A) final body weight, (B) relative weights of white adipose fat tissues (including EWAT, MWAT, or RWAT), (C) relative weights of spleen and pancreas, (D) blood glucose levels, (E) blood glycated hemoglobin (HbA1_C_) levels, (F) triglyceride levels, (G) total cholesterol levels, (H) insulin levels, (I) adiponectin levels, (J) leptin levels, (K) lipid peroxidation (LPO), superoxide dismutase (SOD), (M) glutathione peroxidase (GSH-Px), and glutathione (GSH) contents in liver tissues. #*P* < 0.05, ^##^*P* < 0.01, or ^###^*P* < 0.001 as compared to the control (CON) group; *P < 0.05, **P < 0.01, and ***P < 0.001 as compared to the STZ plus vehicle (distilled water) (STZ) group. All values are means ± SE (*n* = 6 per group). EPE: EPE1: 100, EPE2: 200, EPE3: 400 mg/kg body weight; Glib: glibenclamide (10 mg/kg body weight); Feno: fenofibrate (250 mg/kg body weight). Epididymal white adipose tissue (EWAT); MWAT (mesenteric white adipose tissue); RWAT (retroperitoneal white adipose tissue).

Initially, mice had an average body weight of approximately 18.29 ± 0.11 g. STZ-induced mice (20.40 ± 0.25 g) had significantly lower body weights than the CON mice (21.67 ± 0.45 g) on the 5-day STZ injections (*P* < 0.05). In the end, the STZ group had a significantly lower final body weight than the CON group. Figure 3A shows that in STZ mice treated with EPE, Glib, or Feno, the final body weight did not differ from that of STZ mice. The food intake of STZ mice was greater than that of CON mice (*P* < 0.05). Food intake did not differ between the EPE1-, EPE2-, EPE3-, Glib-, or Feno-treated STZ group and the STZ group (data not shown). The STZ mice had reduced relative weights of epididymal white adipose tissue (EWAT) and retroperitoneal white adipose tissue (RWAT) compared with those of CON mice. The relative tissue weights of the spleen and pancreas did not differ between STZ mice and CON mice (Fig. 3B and C). The STZ mice treated with Glib had a higher relative weight of the RWAT than that of the STZ mice (*P* < 0.001) (Fig. 3D). The relative weights of the EWAT and mesenteric white adipose tissue (MWAT), spleen, and pancreas did not differ between the EPE1-, EPE2-, EPE3-, Glib-, or Feno-treated STZ group and the STZ group (Fig. 3D and E).

### Blood glucose and HbA1c levels

The STZ group had significantly higher blood glucose and HbA1_C_ concentrations than those of the CON group (Fig. 3F and G). EPE1-, EPE2-, and EPE3-treated STZ mice had lower blood glucose concentrations than those of the STZ mice (Fig. 3F). EPE1-, EPE2-, and EPE3-treated STZ mice had lower HbA1_C_ levels than those of the STZ mice (Fig. 3G).

### Plasma triglyceride and total cholesterol levels

STZ mice had higher concentrations of triglyceride (TG) and total cholesterol (TC) than those of the CON mice (Fig. 3H and I). Treatment with EPE1, EPE2, EPE3, or Feno markedly reduced plasma TG concentrations compared with those of the STZ group (Fig. 3H). EPE2, EPE3, or Feno treatment reduced plasma TC concentrations in comparison to those of the STZ group (Fig. 3I).

### Insulin, leptin, and adiponectin levels

The STZ mice had markedly lower plasma insulin concentrations than those of the CON group. EPE1-, EPE2-, or EPE3-treated STZ mice had higher insulin levels than those in the STZ mice (Fig. 3J). STZ mice had reduced adiponectin and leptin concentrations compared with concentrations in the CON mice (Fig. 3K and L). EPE1-, EPE2-, EPE3-, Glib-, and Feno-treated STZ mice exhibited elevated plasma adiponectin levels (Fig. 3K). STZ-induced mice treated with EPE1, EPE2, EPE3 and Feno had markedly higher plasma leptin levels than those of STZ-induced mice (Fig. 3L).

### MDA, GSH-Px, SOD, and GSH analyses in liver tissues

The STZ mice had higher MDA levels in liver tissue than those of the CON group. EPE1-, EPE2-, EPE3-, Glib-, and Feno-treated STZ mice had lower MDA levels than those of the STZ mice (Fig. 3K). The STZ mice had lower GSH-Px, SOD, and GSH levels in liver tissue than those of the CON group (Fig. 3L-N). EPE2- and EPE3-treated STZ mice had higher SOD levels than those of the STZ mice (Fig. 3L). EPE1-, EPE2-, EPE3-, Glib-, and Feno-treated STZ mice had higher GSH-Px and GSH levels in liver tissue than those of the STZ mice (Fig. 3M and N).

### Histology evaluation

**Fig. 4:**
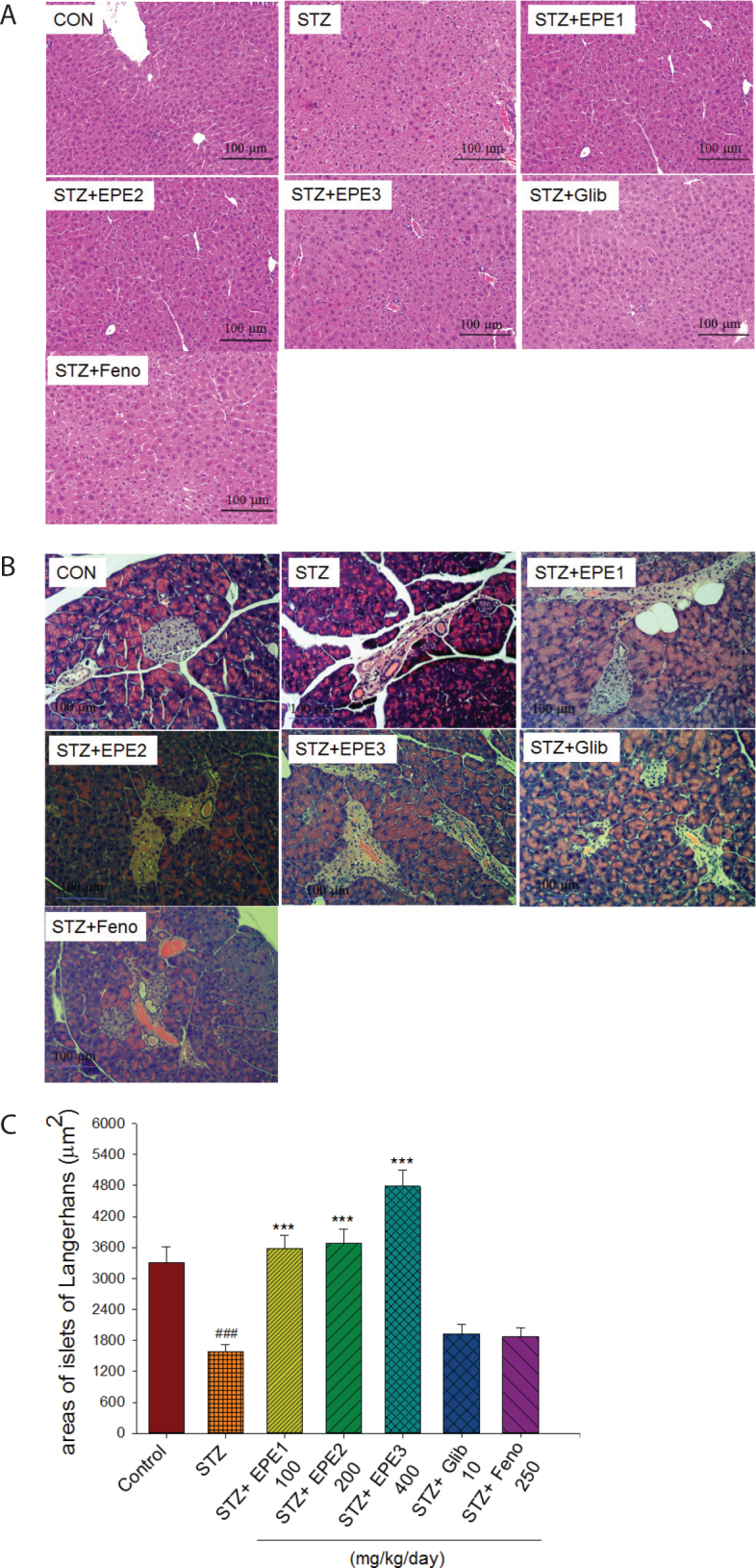

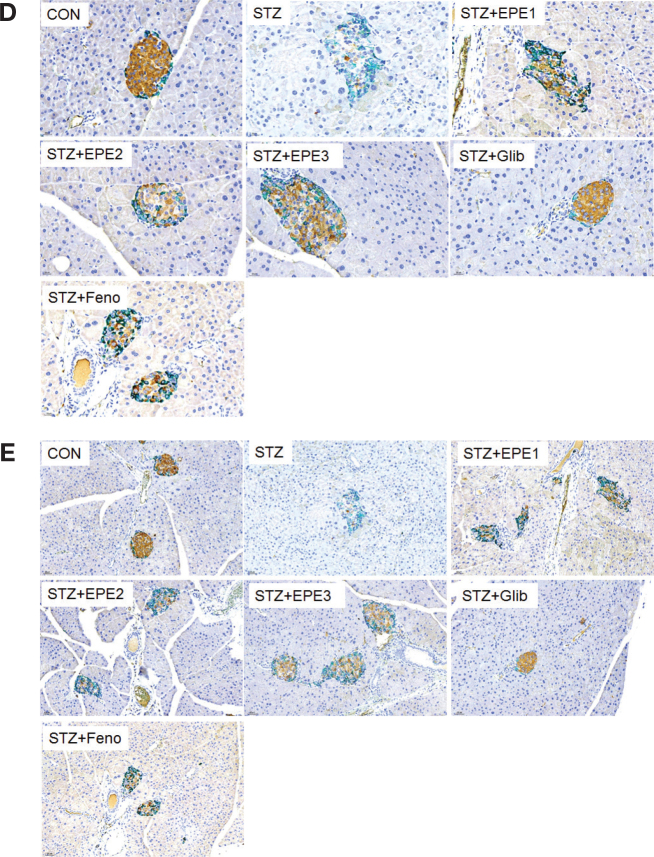
Representative pathogenesis photographs of (A) liver tissues and (B) pancreatic islets of Langerhans, (C) the average area of islets of Langerhans, (D) 200×, and (E) 400× immunohistochemical staining of pancreatic insulin-expressing β cells (brown) and glucagon-expressing (green) α cells of streptozotocin (STZ)-induced mice following treatment with ethyl acetate extracts of *Phyllanthus emblica* L. (EPE), Glib: glibenclamide (10 mg/kg body weight), or Feno: fenofibrate (250 mg/kg body weight). EPE: EPE1, EPE2, and EPE3 (100, 200, and 400 mg/kg body) by hematoxylin and eosin-staining. ^###^*P* < 0.001 as compared to the control (CON) group; ***P < 0.001 as compared to the STZ plus vehicle (distilled water) (STZ) group.

As shown in Fig. 4A, STZ mice exhibited little ballooning of hepatocytes in comparison to that exhibited by CON mice. EPE1-, EPE2-, EPE3-, Glib-, and Feno-treated STZ mice displayed no ballooning phenomenon in comparison to that of the STZ mice. As shown in Fig. 4B, the islets of STZ mice exhibited irregular and less classic round shapes than those of CON mice. EPE1-, EPE2-, and EPE3-treated STZ mice exhibited not only greater islet size but also less deterioration than STZ mice. STZ mice displayed decreased average areas of islets in comparison to areas in the CON mice, and administration of EPE1, EPE2, or EPE3 to STZ mice led to greater islet areas than those of STZ mice; however, Glib or Feno treatment did not lead to differences in areas (Fig. 4C). As shown in Fig. 4D and E, EPE3-treated STZ mice had markedly elevated brown staining (insulin-producing β cells) and reduced green staining (glucagon-producing α cells) at different magnifications.

### Hepatic mRNA levels of target genes

**Fig. 5:**
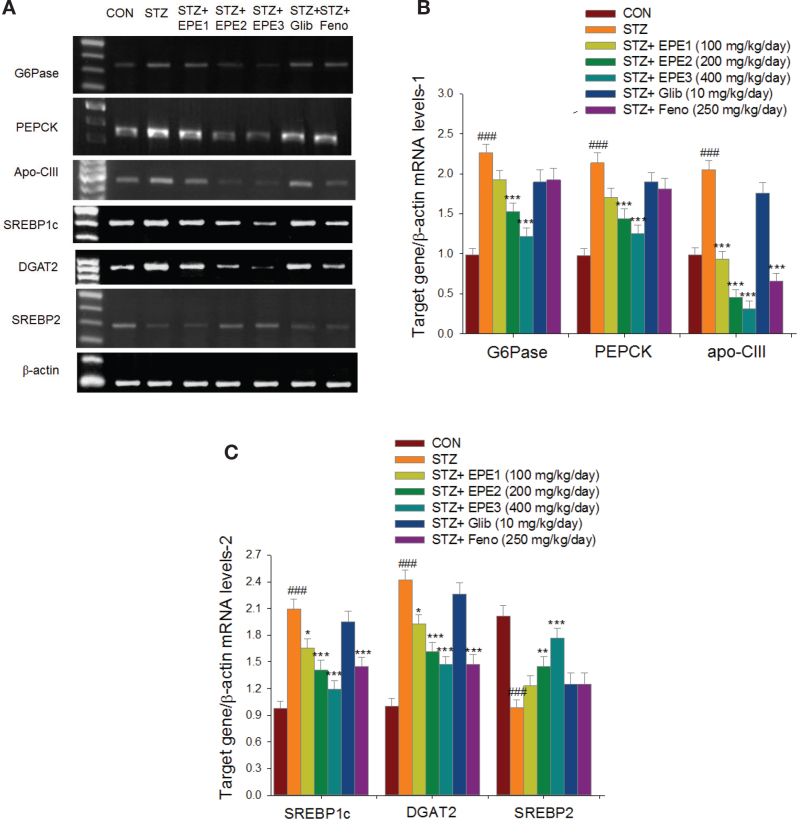
The hepatic target gene mRNA levels in streptozotocin (STZ)-induced mice following treatment with ethyl acetate extract of *Phyllanthus emblica* L. (EPE), glibenclamide (Glib; 10 mg/kg body weight), or fenofibrate (Feno; 250 mg/kg body weight) by semiquantitative reverse transcription polymerase chain reaction (RT-PCR) analysis in STZ-induced mice. EPE: EPE1, EPE2, and EPE3 (100, 200, and 400 mg/kg body). (A) Representative image; (B) and (C) quantification of the ratio of target gene to β-actin mRNA expression. Total RNA isolated from the livers and then reverse transcripted by MMLV-RT, and followed by10 μL of RT products were administered as a template for PCR. The mRNA levels of G6Pase, PEPCK, apo C-III, SREBP1c, DGAT2, and SREBP2 were assessed and quantified by image analysis. Values were normalized to β-actin mRNA expression. All values are means ± SE (*n* = 6 per group). ^###^*P* < 0.001 as compared to the control (CON) group; *P < 0.05, **P < 0.01, ***P < 0.001 as compared to the STZ plus vehicle (distilled water) (STZ) group.

As shown in Fig. 5, STZ mice had elevated PEPCK, G6Pase, apo C-III, SREBP1c, and DGAT2 but reduced SREBP2 mRNA levels in the livers in comparison to those of CON mice. EPE2- and EPE3-treated STZ mice had lower mRNA levels of G6Pase and PEPCK than those of STZ mice (Fig. 5A and B). EPE1-, EPE2-, EPE3-, and Feno-treated STZ mice had reduced mRNA levels of apo C-III, SREBP1c, and DGAT2 in comparison to levels in STZ mice. In comparison to STZ mice, EPE2- and EPE3-treated STZ mice had elevated SREBP2 mRNA levels (Fig. 5A and C).

### Target gene expression in tissues

**Fig. 6:**
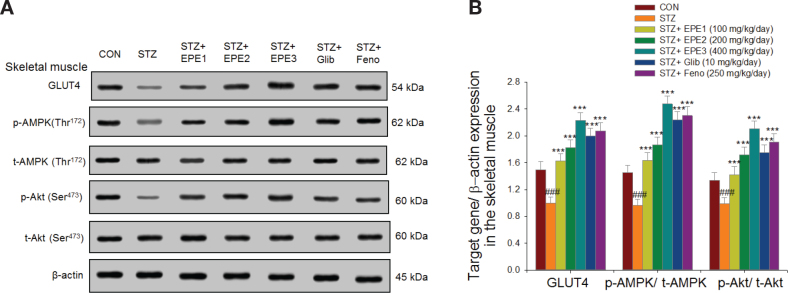
The skeletal muscular target gene expression levels in streptozotocin (STZ)-induced mice following treatment with ethyl acetate extract of *Phyllanthus emblica* L. (EPE), glibenclamide (Glib, 10 mg/kg body weight); fenofibrate (Feno; 250 mg/kg body weight) by Western blotting analysis on membrane GLUT4, p-AMPK (Thr^172^) / t-AMPK and p-Akt (Ser^473^) /t-Akt (Ser^473^). (A) Representative image; (B) quantification of the p-AMPK to t-AMPK and p-Akt (Ser^473^) /t- Akt (Ser^473^). Protein was separated by 12% SDS-PAGE. ^###^*P* < 0.001 as compared to the control (CON) group; ***P < 0.001 compared to the STZ plus vehicle (distilled water) (STZ) group. All values are means ± SE (*n* = 6 per group). EPE: EPE1, EPE2, and EPE3 (100, 200, and 400 mg/kg body).

Figure 6 shows that the membrane GLUT4 expression level was suppressed in the skeletal muscles of the STZ mice in comparison to CON mice. EPE1, EPE2, EPE3, Glib, and Feno treatment dramatically increased membrane GLUT4 expression in comparison to that in STZ mice. The expression of p-AMPK/t-AMPK in the muscles was reduced in STZ mice compared to CON mice (*P* < 0.001). EPE1-, EPE2-, EPE3-, Glib-, and Feno-administered STZ mice had dramatically enhanced expression of p-AMPK/t-AMPK in comparison to that in STZ mice. STZ mice had reduced expression of p-Akt/t-Akt in the muscles in comparison to expression in CON mice. EPE1-, EPE2-, EPE3-, Glib-, and Feno-administered STZ mice had enhanced p-Akt/t-Akt expression in comparison to that in STZ mice (Fig. 6A and B).

**Fig. 7:**
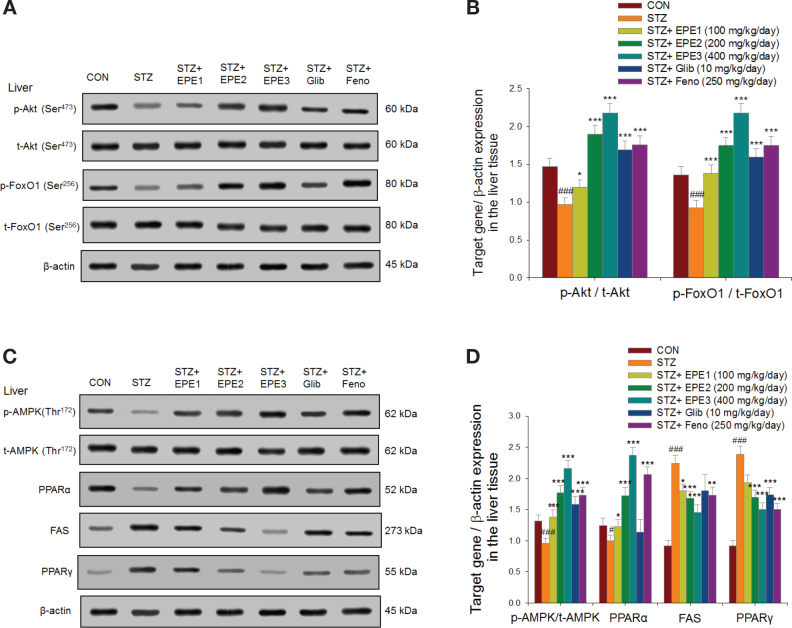
The hepatic target gene expression levels in streptozotocin (STZ)-induced mice following treatment with ethyl acetate extract of *Phyllanthus emblica* L. (EPE), glibenclamide (Glib, 10 mg/kg body weight), or fenofibrate (Feno; 250 mg/kg body weight) by Western blotting analysis. EPE: EPE1, EPE2, and EPE3 (100, 200, and 400 mg/kg body weight, respectively). (A) and (B) p-Akt (Ser^473^) / t-Akt (Ser^473^) and p-FoxO1 (Ser^256^) / t-FoxO1 (Ser^256^), (C) and (D) p-AMPK (Thr^172^) / t-AMPK, PPARα, FAS, and PPARγ. (A) and (C) Representative image; (B) and (D) quantification of the p-AMPK to t-AMPK, PPARα, FAS, and PPARγ. Protein was separated by 12% SDS-PAGE assessed by Western blot. All values are means ± SE (*n* = 6 per group). ^#^*P* < 0.05, ^###^*P* < 0.001 as compared to the control (CON) group; *P < 0.05, **P < 0.01, ***P < 0.001 as compared to the STZ plus vehicle (distilled water) (STZ) group.

Figure 7A and B show that STZ mice had reduced p-Akt/t-Akt expression in the liver in comparison to that of CON mice, and administration of EPE1, EPE2, or EPE3 to STZ-induced diabetic mice increased this expression in comparison to STZ mice. Hepatic p-FoxO1/t-FoxO1 expression was reduced in STZ mice compared to that in CON mice, and administration of EPE1, EPE2, or EPE3 to STZ mice enhanced hepatic p-FoxO1/t-FoxO1 expression compared to that in STZ mice. Figure 7C and D show that in the livers, STZ mice had decreased expression of PPARα and p-AMPK/t-AMPK in comparison to that in CON mice. EPE1-, EPE2-, EPE3-, Glib-, and Feno-treated STZ mice exhibited enhanced hepatic p-AMPK/t-AMPK expression compared to STZ mice. EPE1, EPE2, EPE3, and Feno treatment enhanced hepatic PPARα expression in comparison to expression in STZ mice. Hepatic FAS and PPARγ expression was reduced in comparison to that in CON mice. EPE1, EPE2, EPE3, and Feno had reduced hepatic FAS expression. Treatment with EPE2, EPE3, Glib, and Feno decreased hepatic PPARγ expression in comparison to that in STZ mice (Fig. 7C and D).

### Analysis of the phenolic compounds of EPE

**Fig. 8:**
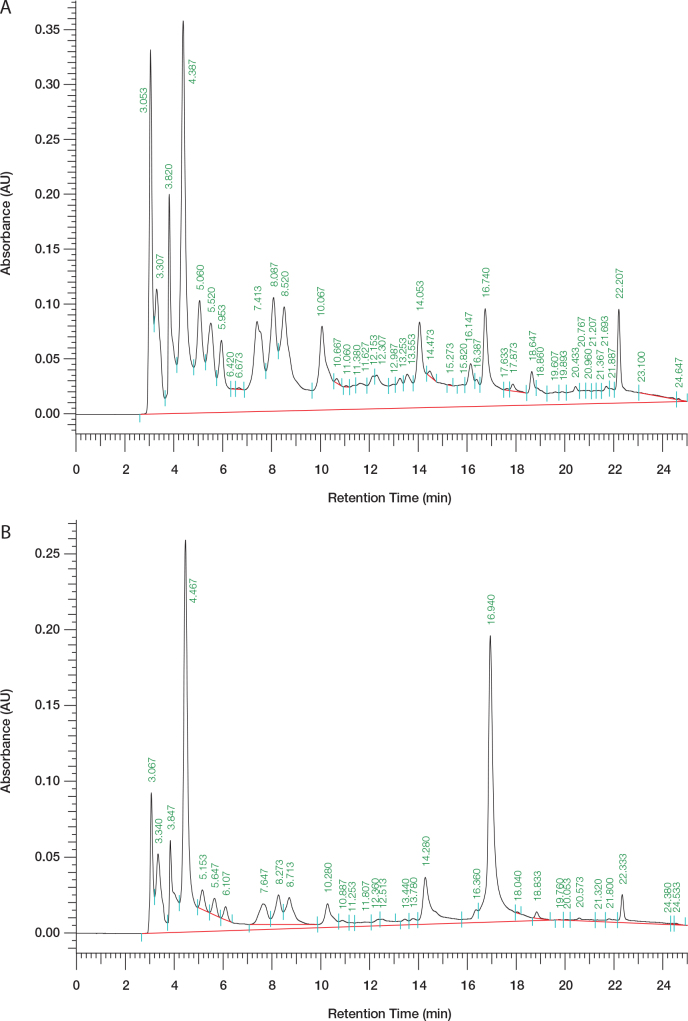
High-performance liquid chromatography analysis of (A) 5000 ppm (24.6 mg/5 mL) ethyl acetate of *Phyllanthus emblica* L., (B) 1000 ppm ethyl acetate of *P. emblica* L. + standard 50 ppm gallic acid + standard 50 ppm chebulagic acid + standard 50 ppm ellagic acid.

This study was designed to examine whether polyphenolic compounds were found in the EPE fraction. The retention times of gallic acid, chebulagic acid, and ellagic acid were 4.567, 14.193, and 16.907 minutes, respectively, as reference compounds to qualitatively and quantitatively analyze the EPE fraction. As shown in Fig. 8, the major absorption peaks of phenolic acids were consistent with the PDA spectra of the reference materials. As shown in Fig. 8 and Table 2, the phenolic compounds found in EPE were gallic acid (3.28%), chebulagic acid (6.44%), and ellagic acid (2.23%).

**Table 2:** Determination of phenolic compounds contained in ethyl acetate extract of *Phyllanthus emblica* L. (EPE) by high performance liquid chromatography (HPLC) analysis

Chemical compounds	Gallic acid	Chebulagic acid	Ellagic acid
Percentage	3.28	6.44	2.23

## Discussion

The aim of the present study was to explore whether EPE displays glucose-lowering and antihyperlipidemic activities in STZ-induced diabetic mice. Taken together, our findings showed that EPE could ameliorate diabetic and hyperlipidemic status in an STZ-induced diabetic mouse model.

Streptozotocin is demonstrated to generate ROS, which contributed to DNA fragmentation and evoked other deleterious changes within the pancreatic tissue (36, 37). STZ-induction could produce ROS and reduced antioxidant enzyme activity especially within the pancreatic tissue, and thus the chemical STZ is adopted to induce diabetic mice to examine natural products whether displaying the antioxidant and preventive activity of STZ-induced oxidative stress (38). In the present study, we found that STZ-induced mice showed increased blood HbA1_C_ levels when blood was exposed to high glucose levels, implying that oxidant damage (39) could be readily observed (Fig. 3E). In the present study, our HPLC data showed EPE contained polyphenols such as gallic acid, chebulagic acid, and ellagic acid. Moreover, this study showed that EPE displays strong antioxidant activities by decreasing MDA and elevating antioxidant enzymatic activities (SOD, GSH-Px, and GSH) in liver tissue and lowering HbA1_C_ concentrations in blood to promote the inhibition of lipid peroxidation, free-radical scavenging, and modulation of lipid and lipoprotein metabolism. Nevertheless, the antidiabetic and antihyperlipidemic activity and underlying molecular mechanism of EPE remain unknown in STZ-induced type 1 diabetic mice. Therefore, we hypothesized that the extracts of EPE would effectively inhibit STZ-induced type 1 diabetes in mice.

Previous studies have shown that the pancreatic degeneration and shrunken islets are regarded as pancreatic oxidative damage (38). Antioxidants exhibited beneficial effects against oxidative stress within the diabetic pancreas (40). Polyphenols belong to one of a variety of antioxidants. Polyphenols are demonstrated to protect cell constituents against oxidative stress and reduce tissue damage by acting directly on ROS or by stimulating endogenous antioxidant defense systems (41). Herbal extracts exhibit protective mechanism against oxidative stress by enhancing antioxidant enzyme activities (SOD and GSH-Px) and averting GSH depletion in STZ-induced diabetic mice (42, 43). Our present HPLC data showed EPE contained polyphenols such as gallic acid, chebulagic acid, and ellagic acid. Our results of administration of EPE to STZ-induced diabetic mice exhibited protective mechanism against oxidative stress accompanied with enhancing antioxidant enzyme activities (SOD and GSH-Px) and were in agreement with results of Wang et al. (43). EPE treatment increased the levels of GSH and these results were in line with a previous study (42). The liver tissue is the most easily attacked organ of the body by free radicals. Liver is a major organ attacked by ROS (44). Since all of the pancreas of mice were collected for morphological analyses, we adapted the liver tissue for the antioxidant activity analysis. The extracts of *P. emblica* have been demonstrated to display antioxidant activity (5, 7–12, 15, 16). Our results on the antioxidant activity of EPE are in agreement with the results of a previous study on the antioxidant activity of *P. emblica* in STZ-induced animals (11). Our results showed that after treatment with EPE in STZ mice, blood HbA1_C_ levels decreased, and histological examination of the pancreas revealed less degeneration and fewer shrunken islets of Langerhans, and increased the size and average area of the islets of Langerhans, suggesting that the antioxidant properties of EPE could improve pancreatic abnormalities.

The β cells generally monitor changes in the availability of glucose. Pancreatic α cells also secrete glucagon, and their secretion is inhibited by glucose. Glucagon plays a deleterious role in T1DM and produces marked glycemia. While insulin levels are low in diabetes, glucagon is present in excess. In this study, EPE-treated STZ diabetic mice had higher levels of pancreatic insulin-expressing β cells but a decrease in the levels of pancreatic glucagon-expressing α cells (Fig. 4D and E), revealing that EPE may function similarly to an insulin secretory agent emanating from the remaining β cells (approximately 30%; this model of STZ-induced diabetic mice with 70% reduction of isle per pancreas (28)) following destruction by STZ and regeneration. These factors provide a therapeutic advantage to T1DM conditions by improving insulin levels after β-cell destruction and/or deterioration of glucagon-producing α cells. In conclusion, the advantage of EPE treatment is due to the recovery of near normal blood glucose and HbA1_C_ concentrations and increased insulin concentrations.

In the present study, HPLC analysis has shown that EPE contains polyphenols components. Polyphenols have shown to modulate hyperglycemia through a variety of mechanisms regarding diabetes mellitus (45). First, polyphenols decreased hepatic gluconeogenesis (45, 46). Second, polyphenols induce adrenergic stimulation of muscle glucose uptake (45, 47), and pancreatic β cell insulin release (45, 48). Our findings are in agreement with previous studies (45–48) showing that EPE-treated STZ mice exhibited not only decreased hepatic gluconeogenesis (including PEPCK and G6Pase mRNA levels), but also enhanced the expression levels of membrane GLUT4 in skeletal muscle, as well as an increase in pancreatic β cell insulin release.

Since the skeletal muscles are quite permeable to glucose and play a role in whole-body insulin-mediated glucose uptake (49), whether the administration of EPE to STZ mice will result in facilitated glucose entry into skeletal muscle and reduced hyperglycemia is an interesting question. Our results demonstrated that STZ mice had lower expression of cell membrane GLUT4 than that of CON mice. The administration of EPE to STZ mice increased GLUT4 expression levels, implying that EPE could facilitate glucose entry. In addition, we next assessed the expression of other critical target enzymes for hepatic gluconeogenesis: PEPCK and G6Pase. A reduction in PEPCK and G6Pase mRNA levels occurred following treatment with EPE and resulted in the suppression of glucose production in the livers.

The methanol extract of *Ecklonia cava* has shown to possess a radical scavenging activity and lowers blood glucose levels and enhances insulin concentration in type 1 diabetic rats by modulating AMPK activation and PI-3 kinase/Akt signal pathways (45), and these findings of EPE’s effects and mechanisms of action were similar to those of the methanol extract (45). Both the insulin signaling pathway and phosphorylated AMPK (phospho-AMPK) have an inhibitory effect on PEPCK and G6Pase transcription (33, 50). Insulin restrains gluconeogenesis *via* Akt-dependent phospho-FoxO1 to suppress PEPCK or G6Pase transcription (33, 51). Through our three results, namely, increased insulin concentrations, elevated expression of p-Akt, and increased expression of p-FoxO1 following treatment with EPE, we observed that EPE enhances insulin levels and increases the phosphorylation of Akt but reduces G6Pase and PEPCK mRNA levels, suggesting that the primary molecular mechanisms of EPE may involve stimulating insulin release from pancreatic β cells. Through effects on insulin-secreting β cells, EPE increases the release of endogenous insulin *via* insulin-p-Akt-p-FoxO1 and/or *via* hepatic p-AMPK to suppress the hepatic PEPCK pathway and improve muscle membrane GLUT4 protein levels, thereby producing hypoglycemia.

Our results showed that STZ-induced diabetic mice exhibited negative growth (Fig. 3A) and that STZ induction in mice had no impact on the relative weights of white adipose tissue (WAT) (Fig. 3B) (which is an expression of the peripheral insulin resistance), indicating that the STZ-induced T1DM model was established. Furthermore, fasting blood glucose levels were markedly reduced in EPE-treated STZ mice, but not Glib- and Feno-treated mice, compared with vehicle-treated STZ mice. Fenofibrate could not improve fasting blood glucose concentrations, and Glib weakly improved glucose intolerance in STZ-induced diabetic mice due to insulin insufficiency. The results presented here suggest that EPE significantly improved fasting insulin levels compared with those of vehicle-treated STZ mice, implying that EPE possesses an insulin secretagogue effect.

In this animal study, STZ induction enhanced plasma TG and TC concentrations, similar to previous studies (52, 53). SREBP2 is known to be involved in hepatic TC synthesis (54). EPE2, EPE3, and Feno treatment of STZ-induced mice were found to be effective in lowering TC levels, implying that the hypocholesterolemic effect of EPE is due to the inhibition of cholesterol biosynthesis through the regulation of SREBP2.

Circulating adiponectin and leptin are involved in fatty acid oxidation and adipose metabolism (55, 56). Blood adiponectin and leptin levels were found to be increased in EPE1-, EPE2-, and EPE3-treated STZ diabetic mice. All the results indicated that EPE suppresses lipid synthesis in the liver but improves fatty acid oxidation by both modulating adiponectin and leptin concentrations and altering blood lipid fluctuations throughout the body (including the liver and/or adipose tissues), thus contributing to hypolipidemia. Leptin replacement has been demonstrated to improve insulin when it is insufficient to help control blood glucose fluctuations in individuals with T1DM (34, 57). Whether EPE treatment is a substitute for insulin therapy for T1DM patients through enhancement of leptin levels remains to be further studied.

On the other hand, polyphenols appear to have an insulin-like effect through different mechanisms, such as AMPK (45). AMPK is a phylogenetically conserved intracellular fuel sensor that regulates fatty acid oxidation and lipid synthesis in most cells (21). PPARα agonists are known to lower TG levels and enhance fatty acid oxidation (31, 32). FAS is associated with the synthesis of fatty acids (58). PPARα ligands lower the expression of the apolipoprotein C-III (apo C-III) gene (59), thereby producing hypotriglyceridemia. In this study, we assessed STZ mice following treatment with EPE to determine how the extract influenced the expression levels of PPARα and FAS and the downregulation of lipogenic genes to reduce plasma TGs. SREBP-1c could enhance the expression of lipogenic enzymes to contribute to the synthesis of fatty acids and the accumulation of TGs (60). DGAT2 catalyzes the final step of hepatic triacylglycerol synthesis (61), and enhanced expression increases triacylglycerol synthesis (62). PPARγ is associated with stimulating adipogenesis and lipogenesis (35, 62). At present, STZ mice have decreased hepatic PPARα but elevated FAS expression and increased apolipoprotein-CIII (apo-CIII), SREBP1c, and DGAT2 mRNA levels. Administration of EPE1, EPE2, EPE3, and Feno to STZ mice enhanced PPARα and reduced PPARγ expression but blocked mRNA expression of the hepatic lipogenic gene SREBP1c, reduced FAS expression, and decreased mRNA levels of the triacylglycerol synthesis genes DGAT2 and apo-CIII to increase the breakdown of hepatic TGs and inhibit hepatic de novo synthesis of TGs, illustrating EPE’s ability to mobilize TGs from liver tissue stores and decrease hepatic TG output, thus decreasing plasma TGs. In the present study, administration of EPE to STZ-induced diabetic mice increased AMPK activation and expression levels of PPARα, but reduced SREBP1c and FAS, apo-CIII, and DGAT2 may at least in part explain the mode of action of EPE amelioration of the blood lipid disorders of STZ-induced T1DM by AMPK/PPARα or AMPK/SREBP1c/FAS observed. Thus, the identification of an activator for AMPK/SREBP1c or insulin signal pathway (Akt) may offer a guideline for the development of a novel product of antidiabetic and/or hypolipidemic agent.

The potential bioactive constituents responsible for the antidiabetic and hypolipidemic activity associated with the re-refraction of EPE were measured by both *in vitro* assays and HPLC analysis, as in our previous study (33), and the results uncovered the fact that gallic acid is the major constituent responsible for the bioactivity of EPE in non-obese diabetes (NOD) with spontaneous and cyclophosphamide-accelerated type 1 diabetic mice. Gallic acid, ellagic acid, and chebulagic acid have been shown to be antioxidants (63). Chebulagic acid has been demonstrated to exhibit antihyperglycemic activity (64). An explanation for EPE’s antidiabetic and antihyperlipidemic activity is that the major ingredients of *P. emblica* fruit extract include the polyphenol components gallic acid, chebulagic acid, and ellagic acid and that these components are responsible for the majority of the antioxidant, antidiabetic, regulation of lipid metabolism, suppression of gluconeogenesis in liver tissue, promotion of membrane glucose uptake in skeletal muscle activity, and mutual restraint of lipid accumulation effects, thus resulting in the preventive activities of EPE on type 1 DM and hyperlipidemia.

More recently, there are newly scientific evidences on physiological and pharmacological effects of *Emblica emblica* extract including the following issues: antihyperglycemic and antihyperlipidemic activity (65), anti-pyretic and analgesic activity (66), antitussive activity (67), anti-atherogenic activity (68), memory enhancing activity (69), spatial learning and memory activity (70), chemopreventive efficacy (71), and immuoregulatory activity including on erythrocyte membranes (72) and our recent publication on T helper cell 1 (Th1) and Th2 in one of T1DM animal model showing that EPE decreased CD4^+^ subset T cell distributions of CD4^+^IL-17 and inhibited the development of autoimmune diabetes by regulating cytokine expression and improvement in the pancreas immunoreactive scores and a decrease in proinflammatory cytokines (33), and anticancer activity (73). Polyphenol is known to beneficial for human health. Curcumin is a herbal components and demonstrates favorable health effects including anti-oxidant and anti-inflammatory, and nevertheless with a poor bioavailability, fast metabolism, and pharmacokinetic profile (74). Additionally, the intestinal microbiota plays a critical role in a variety of diseases, such as diabetes (75). Although the World Health Organization (WHO) recognized the traditional medicine on health care value as a guideline to new drug development (1993), European Food Safety Authority (EFSA) pointed that there is lack of extensive clinical research evidence to establish therapeutic effects (2011). Therefore, further study should shed light on gallic acid’s bioavailability, pharmacokinetic profile, and safety concerns *in vivo*.

**Fig. 9:**
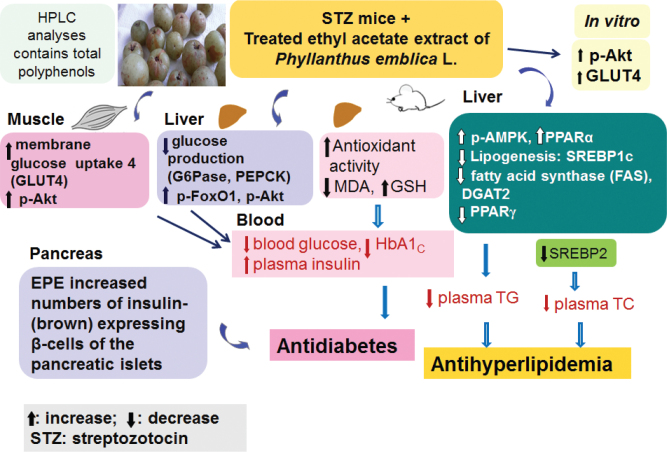
The graphic abstract of ethyl acetate extract of *Phyllanthus emblica* L. (EPE) in streptozotocin (STZ)-induced T1DM mice.

In conclusion, our findings demonstrate that treatment with EPE could not only lower the concentrations of blood glucose, HbA1_C_, TGs, and TC but could also elevate insulin concentrations in the STZ-induced T1DM mouse model (Fig. 9). These data indicate that EPE was effective in lowering glucose but enhancing insulin concentrations, restoring the smaller size, and enhancing the insulin (brown)-expressing β-cells of the islet of Langerhans induced by STZ. This primary antidiabetic mechanism of EPE involves a significant enhancement of insulin-producing β cells (stimulation of β-cell secretion of insulin), thereby producing hypoglycemia. Administration of EPE increased hepatic p-Akt and p-FoxO1 but also decreased PEPCK and G6Pase mRNA levels to inhibit hepatic gluconeogenesis and increased muscle membrane GLUT4 protein levels to improve glucose uptake, thus contributing to the overall antihyperglycemic effect of EPE. Furthermore, EPE (at middle and high doses) reduced the mRNA levels of SREBP2 to lower TC levels. Moreover, EPE treatment enhanced the phosphorylation of AMPK and PPARα but decreased FAS and PPARγ expression, which was accompanied by a decrease in SREBP-1c, apo C-III, and DGAT2 mRNA levels in the liver and hence caused an inhibition of hepatic lipid synthesis, thus contributing to the plasma TG-lowering properties of EPE.
